# Health-Related Participatory Research in American Indian and Alaska Native Communities: A Scoping Review

**DOI:** 10.3390/ijerph16162969

**Published:** 2019-08-18

**Authors:** R. Brian Woodbury, Scott Ketchum, Vanessa Y. Hiratsuka, Paul Spicer

**Affiliations:** 1 Southcentral Foundation Research Department, 4085 Tudor Centre Dr., Anchorage, AK 99508, USA; 2 University of Oklahoma, 5 Partners Place, Stephenson Pkwy, Suite 4100, Norman, OK 73019, USA; 3 Department of Anthropology, University of Oklahoma, 5 Partners Place, 201 Stephenson Pkwy, Suite 4100, Norman, OK 73019, USA

**Keywords:** American Indian, Alaska native, community engagement, participatory research, health research, scoping review

## Abstract

A scoping review was conducted to assess the state of the literature on health-related participatory research involving American Indian and Alaska Native communities. Online databases were searched for relevant articles published between 1/1/2000 and 5/31/2017. 10,000+ data points relevant to community-level engagement in and regulation of research, community research capacity and cultural adaptation were extracted from 178 articles. Community engagement varied across study components: 136 (76%) articles reported community participation in research-related meetings and other events and 49 (27%) articles reported community involvement in initiation of research. 156 (88%) articles reported use of community-level tools to guide or regulate research. 93 (52%) articles reported that community members received research-related training. 147 (82%) articles described some type of cultural adaptation. Across all articles, data points on community engagement were not reported in 3061 (40%) out of 7740 cases. Findings suggest a need for increased community engagement in early stages of the research process and for reporting guidelines for participatory research involving American Indian and Alaska Native communities. There is also need to further existing research on the impact of different components of participatory research on process and outcome measures and to develop funding mechanisms that account for the time and resource intensive nature of participatory research.

## 1. Introduction

Participatory research (PR) is an umbrella term for a suite of research approaches—including participatory action research, community-based participatory research, and tribal participatory research—that share a broad emphasis on balancing generation of scientific knowledge with interventions that benefit communities, promoting community engagement in and shared control over research processes, developing the capacity of communities to participate actively in research, and adapting research objectives and processes to the needs and expectations of participating communities [1,2]. Participatory action research is characterized by a cycle of reflective inquiry undertaken by communities and researchers to inform action for positive social change, equitable power sharing between researchers and researched groups, and an emphasis on community engagement in all stages of the research process [3]. Community-based participatory research is guided by principles that call for equitable relations and co-learning among researchers and communities; development of community research capacity; active community engagement in all stages of the research process; and, interventions that are beneficial, sustainable, and locally relevant [4,5]. Tribal participatory research extends the principles of community-based participatory research to account for the unique political character of AIAN communities by emphasizing respect for the status of federally-recognized AIAN tribes as sovereign nations that interact with the U.S. Federal Government on government-to-government basis and that maintain the right to self-governance and self-determination [6,7,8,9,10]. Tribal participatory research also calls for the use of culturally specific assessment and intervention measures and tribal liaisons to promote communication and coordination between researchers and communities [6,7,8].

PR emerged in response to research approaches that emphasize production of scientific knowledge and are characterized by hierarchical relations between researchers and participants and unilateral control by researchers over research goals, processes, and products [1,3,8,11]. Past health research with AIAN communities often failed to benefit participating communities and sometimes even led to individual- and group-level harm and stigma [12,13]. AIAN people had limited or no control over the objectives and conduct of research or the interpretation and public dissemination of results and research processes did not account for the socially- and culturally-embedded needs and expectations of particular communities [12,14]. By contrast with these research approaches, PR seeks to promote health equity and community empowerment and for this reason is often employed in research involving American Indian and/or Alaska Native (AIAN) communities and other minority populations and marginalized groups that experience social inequalities, political disempowerment, and health disparities [8,15,16,17].

There is a substantial literature on the use of PR to address health-related issues in AIAN communities. However, secondary reviews synthesizing this body of evidence are scarce and the research that does exist is either focused narrowly on specific health conditions or broadly on minority and/or indigenous populations. There is need for secondary research that offers a detailed analysis of how the theoretical principles of PR approaches are applied in practice to a broad range of health-related research involving AIAN communities. Such research could provide AIAN communities and their research partners with strategies for successfully employing PR approaches in community-based, health-related research. This article presents initial results from a scoping review intended to address this need.

## 2. Materials and Methods

### 2.1. Overview of Scoping Review Methodology

Scoping reviews seek to summarize research findings; identify research gaps; determine the need for systematic reviews; and inform policy, practice, and research [18]. In this study, we adhered to the six-step, scoping review methodological framework proposed by Arksey and O’Malley [19]. The steps of the framework are reflected in the organization of the methods section below. In accordance with published recommendations for improving scoping review methodology [20], we also advanced aspects of the framework by requiring two authors to independently review all articles for inclusion in the review, by involving all authors in the development of the data extraction form, and by discussing the implications of our findings for practice, policy, and future research.

### 2.2. Research Team

The authors are staff members of the Center for the Ethics of Indigenous Genomic Research (CEIGR), a National Institutes of Health-funded Center for Excellence in the Ethical, Legal, and Social Implications of Genomic research created to support “culturally grounded comparative research on the ethical, legal, and social implications of genomic research in AIAN communities” [21]. CEIGR and its approach to collaborative research are described in a forthcoming publication [22]. Two authors are of American Indian heritage: SK is a member of the Choctaw Nation and VH is a member of the Navajo Nation and is also Winnemem Wintu.

### 2.3. Research Question and Purpose

This review responds to the overarching question, “What is the state of the scientific literature on health-related PR involving AIAN communities?” We defined health-related research as research designed to assess or promote health. We defined PR as research that employs a specific or general participatory approach. Community-based participatory research, tribal participatory research, and participatory action research are examples of “specific” approaches to PR, while a “general” participatory approach seeks to promote community engagement or participation in research without explicit reference to a particular theoretical framework. In this study, AIAN communities refer to communities of American Indian and Alaska Native peoples located in Alaska and the contiguous U.S. While broad research questions are characteristic of scoping reviews, we also responded to four focused questions aligned with key components of PR:

- RQ1: What is the character and extent of community engagement in research in health-related PR involving AIAN communities?
- RQ2: What tools for guiding and regulating research at the community level are employed in health-related PR involving AIAN communities?
- RQ3: How is cultural adaptation employed in health-related PR involving AIAN communities?
- RQ4: What activities related to development of community research capacity are undertaken in health-related PR involving AIAN communities?

The purpose of this review is to support CEIGR’s work by characterizing the state of the literature on health-related PR involving AIAN communities; identifying opportunities and priorities for future research; and, proposing recommendations for research practice and policy.

### 2.4. Search Strategy

PubMed and PsychINFO were searched for articles published before 1 June 2017 that are tagged with Medical Subject Headings (MeSH) terms or contain keywords related to AIAN populations and health-related PR. Search terms related to PR included: “community based participatory research”, “community engagement”, “participatory action research”, “community institutional relations”, “community participation”, and “action research”. Search terms related to AIAN populations included: “Alaska Natives”, “Inuits”, and “Indians, North American”, “Indigenous, North American”, “Native American”, and “American Indian”. PubMed and PsychINFO searches identified 526 and 6018 articles, respectively. As part of the consultation process, members of the CEIGR steering committee provided 279 articles drawn from their personal libraries. All references were downloaded to Endnote X9 (Philadelphia, PA, USA). After removing 2568 duplicates identified by Endnote, the library contained 4255 unique references.

### 2.5. Article Selection

**Figure 1:**
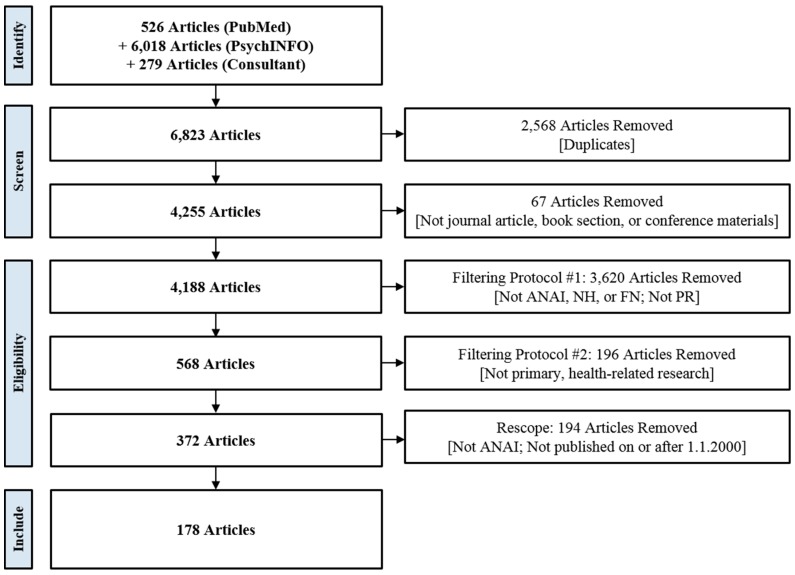
Article Selection Flow Chart.

The selection of articles for the scoping review followed a multi-step process (Figure 1) conducted by RW and SK. After an initial screen that removed 67 documents that were not journal articles, book sections, or conference materials, two protocols were used to determine the eligibility for inclusion of the remaining articles. In the first protocol (Filtering Protocol #1), article relevance to topics, approaches, and populations of interest was determined by sequential review of each article’s title, keywords, and abstract. RW and SK field-tested this protocol on 100 articles, leading to modifications that improved the protocol’s clarity and efficiency. Both RW and SK independently reviewed the 4188 remaining articles. Only articles deemed relevant by both reviewers were retained. 3620 articles were removed, including 60 duplicates not initially identified by EndNote.

The second filtering protocol (Filtering Protocol #2) was used to group articles by type and topic, with the goal of identifying articles describing primary research on health-related topics for data extraction. RW and SK then independently reviewed and sorted the remaining 568 articles. Both reviewers deemed 353 articles eligible for inclusion; only one reviewer deemed 207 eligible. These discrepant articles were reviewed a second time by RW, who made a final inclusion/exclusion decision. In total, 196 articles were removed and 372 articles were retained by the second filtering protocol.

The authors subsequently decided to narrow the inclusion criteria by removing articles that were published before 1 January 2000 or that involved indigenous populations other than AIAN peoples. An additional 194 articles were excluded during this re-scope, resulting in a final library of 178 articles.

### 2.6. Data Extraction

A data extraction form was created to ensure the process of gathering data from articles was standardized, rigorous, and replicable. Development of the data extraction form was an iterative and collaborative process. RW and SK first conducted a review of the search results and the broader literature to identify key elements of dominant PR approaches and to develop queries that captured data relevant to those elements. Two approaches in particular—community-based participatory research and tribal participatory research—were emphasized in the development of the data extraction form. Standardized response sets, inclusion/exclusion criteria, and notes to guide reviewer responses were developed for each query. RW and SK pilot-tested the data extraction form on five articles and then compared results and refined queries, response sets, and supporting notation to promote reliability across reviewers. All authors then participated in a series of conference calls to review and finalize the data extraction form and protocols to guide the data extraction process. The final data extraction form contained 60 queries, 44 of which related to community engagement in the research process. Other queries concerned project purpose, design, methods, setting, and outcomes; use of community resources; community regulation of research; co-learning and capacity building; dissemination of research findings; and, project sustainability. The articles selected for inclusion in the review were divided equally between RW and SK for purpose of data extraction. RW and SK met weekly throughout the extraction process to discuss progress and any questions.

### 2.7. Consultation

Consultation occurred on an ongoing basis throughout the study. From study initiation through the completion of data extraction, RW and SK held weekly conference calls with each other to develop and refine the search strategy; draft study protocols and other resources; compare and discuss the results of the article selection and data extraction processes; review initial results and analysis plans; and, draft and revise manuscripts. In their dual role as coauthors and consultants, VH and PS provided RW and SK with guidance, mentoring, and support in all phases of the research process. Members of the larger CEIGR group also functioned as consultants by providing feedback on study methods and results at reoccurring group meetings.

## 3. Results

Over 10,000 data points were extracted from 178 articles. We report here on a limited set of findings from this dataset that are most relevant to the research questions. We provide a brief overview of the state of the literature on health-related PR involving AIAN communities, followed by detailed results on community engagement in research, community-level guidance and regulation of research, cultural adaptation, and community research capacity.

### 3.1. Overview of Health-Related PR in AIAN Communities

**Figure 2:**
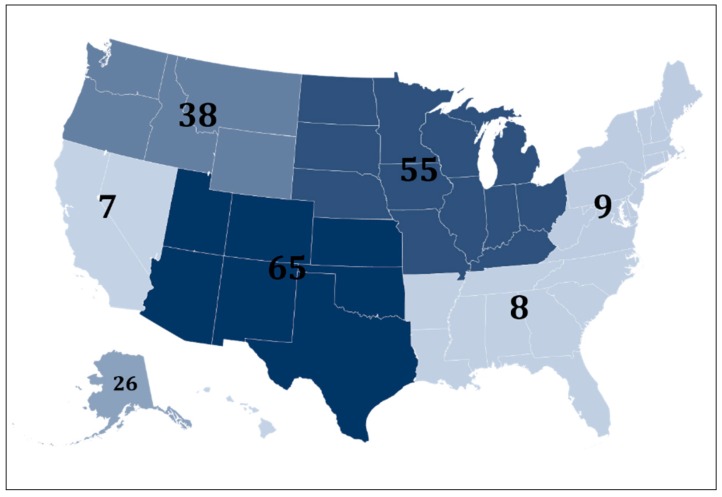
Location by Region of AIAN Communities Participating in Health-Related PR.

Articles described research conducted in AIAN communities across the U.S. (Figure 2). 21 articles did not report the location of the community and 40 articles specified the location of the participating community at the regional level only. Among articles that specified the location of the community at the state-level, states with the highest number of communities participating in research included Alaska (*n* = 26), Montana (*n* = 14), Oklahoma (*n* = 16), Arizona (*n* = 12), New Mexico (*n* = 11), and South Dakota (*n* = 11).

Twenty five (14%) articles were authored by individuals primarily affiliated with tribal governments. By contrast, individuals affiliated with academic institutions were authors on nearly all (*n* = 167, 93%) articles. Authors were affiliated with 66 different academic institutions, 54 of which were listed as an affiliated organization on five or fewer articles. However, some academic institutions appeared to be hubs for PR involving AIAN communities. The University of Washington was the home institution for researchers who authored or coauthored 23 articles. The University of Colorado, the University of Minnesota, the University of New Mexico, Montana State University, and the University of Alaska Fairbanks were associated with 12–16 articles each.

Several articles described research on health conditions that have a documented high prevalence in some AIAN populations, including cancer (*n* = 33, 18%), mental and behavioral health conditions and issues (*n* = 24, 13%), and substance use and misuse (*n* = 34, 19%). 18 (10%) articles described research that evaluated or sought to develop local health care and/or public health systems. Another 23 (13%) articles concerned evaluation and development of health research systems and approaches. More than 1 in 3 articles (*n* = 66, 37%) described research or interventions that addressed the health of children or adolescents and 22 (12%) articles described research or interventions that concerned women’s health. 48 (27%) articles addressed more than one of the health conditions or issues listed in Table 1.

One hundred and three articles (57%) were descriptions or evaluations of the development, processes, or outcomes of a health program. 54 (30%) articles described formative research, including needs assessments and qualitative research into community member perspectives on health-related issues. The use of qualitative data collection methods was common, with focus groups employed in 62 (34%) articles, interviews in 89 (49%) articles, and surveys in 64 (36%) articles. The articles included 20 (11%) self-described case studies/series, 11 (6%) observational studies with a cross-sectional, case-control, or cohort study design, and 12 (7%) experimental trials.

### 3.2. Community Engagement in the Research Process

We assessed the presence of community engagement across 11 components of the research process (Table 2). Data on community engagement often lacked detail or went unreported, with 90% of articles not reporting this information for at least one study component and 54% of articles not reporting on 4 or more study components. Across all articles, information necessary to respond to one or more of 43 queries on community engagement was missing in 3061 (40%) out of 7740 cases.

#### 3.2.1. Initiation

Community members and representatives—including members of community health boards, tribal councils, staff of tribal health services—initiated relationships with research partners from outside the community. For example, one article [34] reports that the board of a local community health clinic approached researchers with a request to study underage drinking in the community and a study of elder care needs in the Eastern Band of Cherokee Indians was initiated when tribal representatives contacted a researcher known to them from previous projects [14]. Other studies were initiated by organizations that advocate for research and policy to promote the health of AIAN populations. For example, the Albuquerque Area Indian Health Board initiated a study to assess the capacity of the public health services available to the Ramah Band of Navajo Indians [35].

The process of study initiation was not always straightforward. Studies sometimes emerged from community review of findings from formative research or discussions between researchers and community representatives about community health needs. One study began after researchers were introduced to community representatives through mutual acquaintances [26]; in another, research activities commenced two years after the principal investigator was introduced by a tribal member to the tribal health director [36]. Researchers also used professional networks and preexisting contacts with AIAN communities to identify communities willing to participate in studies.

#### 3.2.2. Funding

Information on community involvement in funding activities was infrequently reported. Where details were available, community representatives or organizations—including local community health clinics, tribal health administrators and directors, and community members—were described as seeking out, identifying, and recommending funding opportunities [37,38,39,40] and drafting, commenting on, and submitting grant proposals [34,41,42].

#### 3.2.3. Research Question and Study Aims

The extent of community involvement in and control over the determination of research goals was varied across studies. Some articles described communities as leading development of research questions and aims. One article [14] describes tribal representatives as approaching researchers with a defined research question based on identified community needs. In another study, the Cherokee Nation Institutional Review Board (IRB) first rejected a research proposal, and then worked with the investigator to identify research questions that better aligned with community interests [43]. Elsewhere, communities and researchers co-developed questions and aims or modified existing aims on the basis of initial findings. In one study, community elders collaborated with a linguist and historian to determine the goals of a study that explored traditional and commercial uses of tobacco among the Lakota people [25]. After initial findings from a study on environmental lead exposure among AI children living in Northeastern Oklahoma revealed that local non-native children also had elevated blood-lead levels, tribal leaders decided to expand the intervention to include these groups [44]. Finally, researchers sometimes proposed studies that were then approved by community members based on their alignment with community need and cultural appropriateness [35,45].

#### 3.2.4. Administration

Community members in a wide range of roles—including community researchers, tribal agencies, community and lay health advisors, community health aides, and CAGs members—contributed to participant recruitment. Community members also helped with staffing issues. In one study, community members were involved in the selection, interview, and hiring of project staff [26]. Other articles reported that CAGs, leaders, and laypersons community were responsible for identifying individuals to fulfill specific project roles based on their experience and skills [32,46,47,48,49]. Community members contributed to project management by coordinating event logistics; providing translation and information technology services; scheduling and organizing data collection activities; and, facilitating meetings. Communities most often shared administrative tasks with researchers but were sometimes responsible for managing entire studies [50].

#### 3.2.5. Design, Methods, and Approach

Community involvement in the selection or development of the study design, methods, or approach took many forms, including development and selection of data collection methods, interventions, and evaluation plans. Community members also provided guidance on narrower aspects of study design, such as participant compensation and eligibility criteria [51], recruitment strategies [14], and identification of vulnerable populations [47].

Communities were sometimes challenged with identifying study designs that maximized scientific rigor while ensuring that the potential benefits of an intervention were available to the entire community. For example, in communities using control and experimental groups as part of a randomized controlled trial, cross-over or stepped-wedge designs were used to ensure that all participants received the intervention [52,53].

#### 3.2.6. Protocols

Community members contributed to protocol development by developing, revising, and providing feedback on interview guides, surveys and questionnaires, and focus group moderator guides. Community members also co-developed educational materials and training manuals, data sharing rules and agreements, informed consent documents, and research protocols. Community members led the development of culturally and linguistically appropriate data collection tools and procedures. These adaptations were especially important for studies that concerned potentially sensitive issues. For example, one article describes how a group of tribal elders and council members, as well as parents and adolescents from the community required interview protocols to include “safety valves” that allowed AI youth being interviewed about domestic abuse and suicide to opt out of specific questions and to be informed about or referred to relevant health services [54] (pp. 6–9).

#### 3.2.7. Meetings and Events

Meetings were used early in the research process to discuss potential collaborations between researchers and the community, to build relationships, to train co-researchers hired from within the community, and to develop project components. In the later stages of a study, meetings served as an opportunity to obtain community member feedback and to disseminate research findings results of research.

Meetings most often occurred between the research team and CAGs, but also involved tribal officials or representatives from tribal government offices or organizations, including tribal leaders, tribal health directors, tribal health organization staff, and tribal councils. Meetings between the research team and either the broader community or project staff hired from within the community were infrequently reported.

#### 3.2.8. Data Collection and Analysis

Community members conducted interviews, facilitated focus groups, and administered surveys. They also took notes during research-related meetings, collected demographic information, and validated and recorded surveillance data. Involving community members in these activities had practical benefits. For example, participants may be reticent to discuss culturally-sensitive issues with strangers or more likely to participate in a study if they are recruited by trusted colleagues, and employing interviewers who are fluent in a local language helps ensure studies account for a broad range of community perspectives [29,55].

Community members assisted with the qualitative analysis of interview and focus group transcripts by developing and revising codes and codebooks, coding and review of coded transcripts, identifying themes, and verifying summary results. Other analysis activities included evaluating results from previous research, identifying the need for specific analyses, and analyzing survey results.

#### 3.2.9. Intervention

Community member contributions to intervention development and implementation varied with project objectives. A project to address barriers to diabetes care in an AI community in the Pacific Northwest leveraged the unique skillset of a community member who owned a farming business to construct a community garden that provided fresh produce at no charge to community members [56]. Other articles reported that community members helped develop tools to test predictive factors for promoting sobriety [55], identified steps to prevent lead exposure [44], and drafted rules to guide ethical research in indigenous communities [57].

Community members frequently collaborated on efforts to develop or adapt educational materials and curriculums. In one study, community members participating in a study to test the effectiveness of a program to increase rates of organ and tissue donation among AI populations recommended that the curriculum be revised to include storytelling components and an emphasis on cultural values [32]. In another study, community members reviewed and revised a culturally informed child safety curriculum [52], tailored cancer education workshops and smoking cessation programs to align with community needs and values [58,59], and created posters to prevent lead exposure and videos to promote cancer awareness [60,61].

#### 3.2.10. Dissemination

Community members drafted manuscripts [62] and CAGs, tribal councils, and other community groups were involved in reviewing, editing, and approving manuscripts for publication [63,64]. Community members also developed dissemination plans [65] and reviewed and provided feedback on research results and dissemination products [44,49]. Finally, community members presented research results at local community events and at national scientific conferences [14,34] and hosted and attended community dissemination meetings [66,67].

### 3.3. Community Guidance and Tribal Regulation of Research

We assessed articles for descriptions of community-level mechanisms for guiding or regulating research (Table 3). A majority of articles (*n* = 125, 69%) described the use of CAGs and the role of tribal liaisons/facilitators and tribal councils in regulating research was described in 64 (36%) and 45 (25%) articles, respectively. 17 (9%) articles described the use of tribal resolutions in guiding or regulating research, while other regulatory documents were less frequently described. Overall, the use of mechanisms for regulating research was frequently reported: 156 (88%) articles described the use of at least one mechanism for guiding or regulating research and 123 (68%) of articles described the use of two or more regulatory mechanisms.

#### 3.3.1. Community Advisory Groups

CAG membership was diverse and included parents; youth; tribal elders; tribal law enforcement; researchers; clinicians; patients; faculty and students of tribal colleges; experts in local languages and culture; members of tribal and intertribal councils; members of tribal health boards and major tribal agencies; and, community members with relevant interests and experiences. In some cases, preexisting community or local government groups—including youth councils and groups, local advisory boards, health advisory committees, and governance bodies within tribal health organizations and AN regional corporations—fulfilled the advisory and research review functions of a CAG [34,66]. CAGs overseeing research involving multiple communities included representatives from each of those communities [44]. It was also common for CAGs to include members with skillsets and experiences relevant to the nature of the research project. For example, a study to test a culturally adapted curriculum to increase participant in organ donation programs was guided by a CAG that included the executive director of a regional organ bank [32]. CAGs usually comprised 25 members or fewer [16,44,68,69], but one article [70] reported that a CAG representing all AI tribes and urban associations in North Carolina grew from 25 to 190 members over the course of 6 years. CAGs tended to convene monthly during key phases of the study, or as needed for project initiation and results dissemination [48,71].

CAG roles and activities were highly diverse and included tasks related to study development and management; interpretation and communication of study results; and, the review and approval of research proposals and products. CAGs contributed to study development by identifying community needs and research aims and questions and providing input on study design and content. CAGs also helped draft research protocols, sampling plans, surveys and other data collection instruments, focus group moderator guides, and intervention manuals. CAGs fulfilled management and administration responsibilities within research projects by selecting recruitment sites and identifying study participants; building community support and nominating cultural advisors; conducting recruitment and consenting participants; implementing interventions; providing input on issues related to staffing and finance; and, handling personnel challenges. CAGs played a critical role in providing community perspectives on findings and helping to develop and present findings to the community and other stakeholders. For example, CAGs were involved in interpreting results; writing manuscripts and analyzing data; reporting results to the community and to representatives of tribal government; and, participating in dissemination activities. CAGs were also engaged in the review and approval of study protocols and products, including project proposals and grant applications, data collection and recruitment protocols, and research publications and presentations. In this role, CAGs were often tasked with ensuring that project instruments, protocols, and processes were culturally and linguistically appropriate.

Several articles described CAGs as fulfilling multiple roles and contributing to projects throughout the research process, from identification of research aims to dissemination of results [48,72,73,74]. For example, as part of a multi-site study assessing substance abuse issues of concern to three AI communities, CAGs contributed to the “development of the research questions and data collection tools, recruitment and interviewing, analysis of the data, and interpretation and sharing of the results” [48] (pg. 163). CAG roles sometimes expanded beyond the scope of a single study. One article describes how a 12-member CAG initially convened to address the high rate of diabetes in their community first developed and implemented a culturally adapted version of the Stanford Chronic Disease Self-Management Program before going on to secure funding for ongoing diabetes prevention efforts in three nearby communities, partner with the Indian Health Service (IHS) to serve as consultants on health promotion efforts in other AI communities, and function as advisors for a national conference on youth obesity [16].

#### 3.3.2. Tribal Mechanisms for Regulating Research

Multiple individuals and groups contributed to community-level regulation of research. Regulation by tribal councils was reported in 45 (25%) articles, while unspecified tribal and community leaders were reported in 11 (6%) and four (2%) of articles, respectively. Other individuals and groups in leadership roles that were involved in research regulation included tribal boards of directors, tribal governors, tribal chiefs, the executive administrations of tribal nations, administrative and cultural oversight committees, and tribal council representatives. In some cases, committees and boards responsible for issues specific to health or research reviewed and approved research. These included research review boards; tribal health boards and committees; tribal health departments; and, research review committees operated by tribal health organizations. Responsibility for regulating research also fell to organizations working in an area relevant to the aims of a given study. For example, a project that included the development of a community garden near an elder care facility in order to promote access to healthy food options sought approvals from tribal programs related to agriculture, housing, elders, and nutrition [56]. Similarly, a study to test a theoretical model for prevention of alcohol misuse and suicide risk among AN youth was approved by superintendents and school boards at each of the participating schools [75]. Research was reviewed and approved by IRBs affiliated with tribes, Indian Health Boards, IHS, and academic institutions. Use of tribal IRBs was reported less frequently than was use of IRBs affiliated with the Indian Health Service (18 articles (10%) vs. 23 articles (14.4%)) or a partnering institution, but more frequently than the use of IRBs affiliated with an Indian Health Board (18 articles (10%) vs. three articles (2%)). These individuals and groups were involved in activities similar to those undertaken by CAGs, including oversight of research projects and development, review, and approval of research proposals and products.

Comparatively few articles described the specific processes and documents used to regulate research. 17 (9.4%) of articles explicitly reported the use of tribal approvals, reviews, or resolutions. Other common processes and documents included data sharing agreements; research protocols, codes, and agreements; and, memoranda of agreement/understanding. These regulatory documents were sometimes used to guide the overall approach to research or to delineate rules and expectations within a specific phase of the research process. One article describes a research protocol that lists 15 requirements for research proposals, including stipulations related to participant protections; community-researcher interactions; CAG functions; data management; prohibited conduct; and, community rights [37]. Another article reports the use of a publication protocol that outlines the process for involving community members in the development of research products and the dissemination of results to stakeholders within and outside the community [67].

It was very common for communities to employ multiple mechanisms for regulating research. For example, in a study on elder health care needs involving the Eastern Band of Cherokee Indians, formal approval in the form of a tribal resolution was sought from the Tribal Council, the Tribal Elder Council, and the tribal IRB, while the tribe’s Health and Medical Board was appointed to fill the role of a CAG. In addition to these internal protections, the community also sought the approval of the IRB affiliated with the PI’s academic institution [14]. A project to prevent cardiovascular disease sought and received approval from the tribal council, as well as a regional IHS IRB, the IRB affiliated with the research group’s academic institution, and a national protocol review committee [37].

Articles describing multi-site studies reported that approvals were sought from each of the participating communities. A study of chronic disease among AN people sought and received tribal resolutions from all of 26 AN communities participating in study, as well as approvals from the Alaska Area IRB, the national IHS IRB, tribal health research committees, and the governing boards of participating tribal health organizations [39]. Authors of another article reported seeking approval from tribal research review and advisory boards affiliated with each of the 11 communities involved in a randomized control trial to test a home-visiting intervention to reduce health risks for young AI mothers and their children [47]. Different communities participating in a multi-site study sometimes elected to employ different regulatory measures. For example, among four tribal communities involved in a study on substance use, three communities made use of the IRB affiliated with the research group’s academic institution and one chose to rely on an IHS IRB for research review [76].

### 3.4. Cultural Adaptation

We assessed whether specific components of a research study or project—including study design, study protocols, interventions, and dissemination methods and products—were culturally adapted (Table 4). Adaptation of study design was reported in 72 (40%) articles. Adaptation of study protocols—including protocols related to study or project administration, recruitment, and data collection and analysis—was reported in 109 (61%) articles. Adaptation of interventions and of dissemination methods was reported in 80 (44%) and 35 (19%) articles, respectively. Cultural adaptation was common, with 146 (82%) articles reporting adaptation of at least one study component and 91 (51%) articles reporting adaption of two or more components.

#### 3.4.1. Study Design

Adaptation of study designs included modifying the design of experimental trials to maximize potential benefits for or avoid harms to participants. For example, in a randomized controlled trial to assess the impact of an intervention to reduce health risks for AI teen mothers and their children, community members advocated for the use of an “optimized” variant of standard care in the control group in order to better address the health needs of participants randomized to control ([47], p. 506). Another trial used a wait-listed randomized design to ensure that all participants eventually received the intervention [77] and an intervention to prevent underage drinking among AI youth was designed to involve all students in the participating communities in order to prevent stigmatization that could result from singling out AI youth for intervention [26].

#### 3.4.2. Study Protocols

Study protocols related to data collection and analysis was frequently adapted in ways that accounted for cultural norms and expectations related to conceptions of data and methods of data collection. For example, talking circles were used as a culturally adapted technique for collecting data from semi-structured group discussions [78] and stories and songs were identified as culturally relevant types of data [79]. In one study, researchers used data collection measures previously validated in an AI population [14]. In another study, researchers adapted data collection instruments to account for cultural norms of the participating community [42]. Studies also incorporated community practices into data collection activities. For example, providing food and sharing gifts at public gatherings is a common practice in many AIAN communities and researchers acknowledged these expectations by offering food and culturally specific gifts to participants [80].

In some cases, males or females conducted data collection exclusively in order to respect cultural sensitivities related to specific health issues. For example, in a study on male sexual health, male tribal members were selected for training on interviewing techniques and protocols [81] and female tribal members received two-day training as lay health advisors as part of an effort to improve community awareness of cervical health issues [82]. Other adaptations to data collection and analysis protocols sought to account for community perspectives by involving community members in the analysis of qualitative data [31] and in the development of focus group, interview, and survey questions that were culturally appropriate, comprehensible to participants, and relevant to community needs [34,65,83]. Recruitment protocols were adapted to better leverage existing social networks within participating communities by utilizing snowball sampling techniques and local media to raise awareness of a study and by conducting recruitment at powwows, health fairs, and other community events [29,84,85].

#### 3.4.3. Interventions

Cultural adaptation of interventions often involved incorporation of traditional community practices or concepts into interventions. For example, one study combined training in videography with the tradition of storytelling common among AN cultural groups to increase knowledge and awareness of cancer among rural AN communities [60]. A culturally tailored website that distinguished between culturally appropriate uses of tobacco (e.g., ceremonial pipe-smoking) and tobacco abuse and addiction was developed for a smoking cessation study [86]. A study on the needs of AIAN youth transitioning into adulthood incorporated the community’s concept of the medicine wheel into a holistic approach to case management [87]. In another study, tribal elders worked with researchers to design a culturally appropriate playground that included traditional housing structures and enabled children to engage in subsistence activities [33].

#### 3.4.4. Dissemination Methods and Products

Adaptations of dissemination methods and products included use of local media and culturally relevant imagery to communicate study results. In one study, information related to the evaluation of a dating violence prevention program was disseminated through several local media outlets, including radio, cable television, and newspapers [42]. In another study, dissemination of research findings involved data visualizations that used images drawn from local culture [88].

Specific types of cultural adaptation, including the use of local language and accounting for the special role that elders play in many AIAN communities, were described in several articles and applied to multiple study components. Incorporating local languages into research processes and products was one of the most common types of cultural adaptation. Data collection activities [80,83], interventions [89,90,91], and dissemination of research findings [88] were adapted to account for the languages used by community members. For example, one article described using the Hopi language in a digital storytelling intervention [92] and another described conducting focus groups in the Navajo language and developing a cancer health education intervention that was linguistically appropriate [80]. In a study on the health effects of stress and associated coping strategies in a rural AN community, preliminary findings were shared in the local language at open community meetings [88].

Several studies accounted for the role that elders play in AIAN communities as venerated, knowledgeable leaders. One study conducted focus groups in Navajo for the express purpose of encouraging participation by elders [80] and other studies explicitly sought input from elders [93,94]. Other studies recruited elders as members of the research team, where they filled various roles, including mentoring participants in healthy behaviors [95], serving as a tribal outreach coordinator [32], and reviewing study findings [87]. Elders serving on Elder councils and CAGs helped evaluate and approve proposals [14,34]. Researchers also adapted study protocols to account for the impacts of elders on community participation. For example, one study organized focus groups by age so that younger participants, who may have been reluctant to speak before or against their elders, were more likely to engage fully in the discussions [96]. Another study purposefully involved elders in the intervention in order to legitimate the project in the eyes of the community and thereby to encourage broad community participation [97].

### 3.5. Capacity Building and Co-learning

We asssessed whether studies described or promoted community research capacity or co-learning among researchers (Table 5). To characterize the state and development of community research capacity, we searched for descriptions of research-related trainings and paid employment opportunities for community members, as well as descriptions of prior experience with research among community members. To characterize the state and development of co-learning among researchers, we searched for descriptions of researchers receiving training in PR or in community culture and for text stating that researchers had prior experience with the participating community. 93 (52%) articles reported that community members received general research training and 43 (24%) reported that community members were trained in PR. 60 (33%) articles reported that researchers received training in community culture or related topics and 47 (26%) articles reported that researchers had or received training in PR. A large number of articles did not report information related to community research capacity and co-learning. For example, 135 (75%) articles did not report whether community members received training in PR and 117 (65%) articles did not report whether researchers received training in community culture or related topics.

#### 3.5.1. Developing Community Capacity to Regulate Research

Efforts to increase community capacity to regulate research included development of tribal and community-based mechanisms for regulating and guiding research. Articles described the development of memoranda of understanding/agreement [98], guiding codes and principles for research [62,99], and CAGs [89]. Whether these resources were appropriate for or used in subsequent research was unreported in most articles. Other articles described development of tribal IRBs [50] and training CAG members in IRB research review processes [100] and research ethics [14].

#### 3.5.2. Developing Community Capacity to Conduct Research

Capacity building also took the form of training community members in skillsets and knowledge relevant to research. This included training in collecting and analyzing data moderating focus groups and conducting interviews and surveys; consenting participants; and, providing home health visits. Some studies provided multiple training opportunities for community members. In one study, community members were trained to function as recruiters; data collectors; consent coordinators; data managers; artists; and, outreach coordinators [101]. In another study, researchers offered training in GPS and GIS mapping, photography, and presentation skills [71]. For an investigation into health disparities and toxicant exposure among Akwesasne Mohawk young adults, researchers offered community members training in anthropometry; phlebotomy; cognitive assessment; nutritional surveys; and, interview skills [102].

Trainings could be intensive. One study involved more than 80 h of community member training in human subjects research protections and intervention delivery for individuals who functioned as family health educators [47]. In another study, AI teenagers participated in a 6 week-long training course on videography culminating in the development of an asthma awareness video [65]. For a project to promote awareness of cancer and cancer treatment options among AI populations, community members were trained as patient navigators responsible for guiding patients through the continuum of care related to cancer treatment. Navigators received 125 h of initial training, 35 h of additional training through site-specific education and national seminars, and 40 h of semi-annual updates [103].

#### 3.5.3. Paid Employment

Community members were hired and paid as translators; administrative assistants and community coordinators; community research associates; project coordinators; community organizers; liaisons responsible for planning fieldwork; home visitors; and, research staff members. In one study, lay health advisors responsible for communicating with community members about issues related to cervical health received stipends and other community members were hired in supervisory and data collection roles [44]. In another study, AI individuals were hired to fill key project roles, including study coordinator and physician [64]. Community members sometimes saw their contributions as gifts for or responsibilities owed to the community and refused payment for their efforts [33].

#### 3.5.4. Researcher Co-learning

Researchers developed knowledge of community culture and needs and expectations regarding research through direct and sometimes extended engagement with communities. Researchers reported having between 2 and 10 years of experience participating in and establishing relationships with a community prior to the start of a study [36,47]. One article states that “prior to initiating focus groups, our research team spent over a year establishing relationships with tribal members” [37] (pp. 123). The authors of one article volunteered at community events and visited the community several times prior to research [104]. Another researcher describes seeking out and cultivating a mentoring relationship with a community elder in order to better understand the community’s culture and needs [16].

These informal methods of developing relations and knowledge of communities were sometimes supplemented by cultural competency trainings provided by communities or through research activities designed to develop relationships and promote trust. In one study, researchers received training about local culture and historical trauma [71]. In another study, researchers gained knowledge through meetings held for the express purpose of building relationships [105]. Researchers also learned about PR approaches. In one example, exposure to community-based participatory research led the investigators to completely rework their research protocol [28].

## 4. Discussion

The purpose of this scoping review was to characterize the state of the literature on health-related PR involving AIAN communities and to propose recommendations for research in this area. Below, we describe several opportunities for conducting future research, improving research practice, and amending research policy to better support health-related PR involving AIAN communities.

### 4.1. Research Recommendations

While the literature on PR involving AIAN communities is remarkably broad in terms of the range communities, health issues, and interventions it describes, several research areas have yet to be explored and some important research questions remain unaddressed. In particular, there is need for research that assesses how different PR approaches and components of approaches interact with community-level factors to affect study processes and outcomes. Although past scholarship has shed light on how different components of dominant PR frameworks contribute to research outcomes [106,107], much remains to be determined about the interrelationships between factors related to research processes and outcomes and the characteristics of community characteristics participating in research. Potential research questions include:

- How do community values and norms, as well as community resources and infrastructure, affect the acceptability and feasibility of different study designs and objectives?
- Do various PR approaches differentially affect project timelines and costs, participation and retention rates, or the effectiveness of an intervention?
- How do different strategies for adapting PR approaches and evidence-based interventions to the needs of individual communities compare to one another in terms of ease of application and impact on outcomes?
- What is the perspective of community stakeholders on the costs and benefits of different PR approaches and the relative importance of the various components of those approaches?

There is also need to increase the variety and quality of study designs used in PR involving AIAN communities. Over half of all articles (103, 57%) involved descriptions or evaluations of the process or outcomes of community health programs. In most cases, program evaluations did not include process or outcome measures that would allow for formal analysis of program effectiveness. By contrast, only 23 articles (13%) describe observational studies and experimental trials were quite limited. These types of research are crucial to designing successful community health programs and for identifying factors that promote or undermine health.

### 4.2. Practice Recommendations

Several opportunities for improving PR research practices exist. Key among these is the development of standardized guidelines for reporting on the use of PR approaches in health research involving AIAN communities. As described above, the reviewed studies often failed to report basic information about the nature and extent of community engagement in the research process. This observation reflects the trend of inadequate reporting in the broader health research literature [108]. Incomplete, inconsistent, and inaccurate reporting is a waste of research funding and can detrimentally impact subsequent research and clinical care [109,110]. Reporting guidelines have potential to improve the quality of health research reporting and for this reason have been offered as a strategy for addressing documented deficiencies in how health research is reported [108,110].

Guidance on how studies that employ PR approaches should be conducted is widely available. Scholars have proposed general principles to guide the conduct of PR [5,111,112], organizations representing the interests of AIAN people have developed or collected guidelines and other resources for conducting PR in AIAN communities [113,114], and many of the articles reviewed in this study included practice recommendations based on “lessons learned” from prior research [17,115,116,117,118].

By contrast, guidelines for reporting on PR in the context of AIAN communities do not exist, although resources that could aid in the development of such guidelines are available. For example, articles that provide guidance for reporting on community-based participatory research have been published [119,120] and the EQUATOR Network database currently contains one set of best practices for reporting on participatory action research [121]. Efforts to develop standards, guidelines, and/or recommendations for reporting on PR could initially scaffold off these and other resources—including the principles and mechanisms that inform dominant PR approaches—in order to develop a set of flexible expectations for reporting on PR that allow for critical appraisals of and comparisons across studies while accounting for variation in research goals and community needs. Multiple stakeholders, including representatives of community-based organizations and academic institutions, should be involved in drafting these documents. Organizations that represent the interest of AIAN communities as they pertain to research practice and policy (e.g., National Congress of American Indians, National Indian Health Board), as well as those that are practiced at supporting collaboration between communities and researchers (e.g., Campus-Community Partnerships for Health, CDC’s National Community Committee) are well-positioned to lead these efforts.

Our findings also suggest areas for improvement in the conduct of PR involving AIAN communities. First, participation of community members in the research process varied substantially by study component and were lowest for activities related to study initiation and selecting and securing grant funding. Given the emphasis in PR on building research questions and objectives around the needs and interests of communities, there is need to promote the ability of communities to proactively identify research opportunities and to initiate relationships with researchers. Second, only a small proportion of articles (<10%) reported the use of any of four types of documents for regulating research. Use of regulatory documents can give stakeholder groups a shared language for discussing these and other complex issues and can promote trust and accountability by clarifying and making enforceable the terms of a research partnership. Finally, less than 15% of articles listed authors whose primary affiliations were to tribal governments or community-based organizations. Direct participation in development of scientific manuscript contributes to community research capacity and supports shared control over interpretation, presentation, and public dissemination of research results. This issue is particularly important for AIAN peoples, who have experienced harm consequent to release of stigmatizing research results.

### 4.3. Policy Recommendations

Numerous articles stated that the resource-intensive nature of PR posed a barrier to the adoption of these approaches by researchers and communities alike. For example, building relationships with communities and engaging them meaningfully in research processes required more time and funding than was usually available through research grants. Articles also described how the emphasis in PR on these long-term processes is at odds with the imperative in academic research culture to rapidly complete research projects and to publish results early and often. The complex and multilayered review processes common in research involving AIAN communities furthered concerns about costs and timelines in the context of PR. Researchers acknowledged the value and legitimacy of these mechanisms but described the administrative burdens they imposed on researchers as potential disincentives to conducting research with AIAN communities.

PR is supported by major funders of health research, including NIH and the CDC [122], and has been identified by the National Academies of Sciences as an essential competency for public health professionals [123]. These commitments to community engagement could be demonstrated through development of grant funding mechanisms and opportunities that better support the unique needs of PR. As a first step, a greater proportion of all funding opportunity announcements at the NIH could be earmarked for PR projects and more requests for proposals could include utilization of a PR approach in the eligibility criteria. The budget section of grant applications could be modified to allow requests for supplementary funding for efforts to undertake activities in support of community engagement in research processes. Similarly, timelines for projects could be expanded to account for time spent building relationships, navigating community-level review processes, consulting with CAGs, hiring and training community members, and developing and disseminating results within the community.

Although articles frequently acknowledged the benefits of PR for communities, recognition of the scientific value of PR was less common, despite its role in improving the efficiency, rigor, acceptability, and feasibility of research. For example, community members can help researchers preemptively identify and address logistical barriers to participation, thereby increasing the efficiency of the recruitment process. Scientific rigor is similarly improved by leveraging community knowledge to account for factors that could negatively impact retention rates and the representativeness of the study sample. Tailoring programs and interventions to account for the capacities, resources, needs, and goals of communities promotes the feasibility and acceptability of research, but also provides evidence and strategies for addressing challenges in translational research, such as identifying processes for adapting evidence-based interventions. PR also benefits science by opening doors to research with underserved or marginalized communities and by bringing underrepresented perspectives into the scientific community.

Raising awareness among researchers of the scientific benefits of PR is crucial to arguing for its broader use in community-based research and could be achieved through coordinated intervention by stakeholders in research. For example, academic institutions and programs that produce scholars working in health research could increase access to training in PR theory and practice; develop programs, certifications, and extracurricular workshops and seminars on PR; and, include competency in PR methods as a requirement for any health research degree. Funders of research could make use of PR approaches a condition of funding for community-based research, especially in instances where proposed research would involve marginalized or otherwise disempowered communities. Journals that publish articles on community-based health research could require descriptions of community engagement processes and encourage or require the use of PR approaches as a condition of publication. AMA, APHA, and other professional organizations whose membership includes health researchers could promulgate guidelines or recommendations that advocate for the use of PR approaches in community-based research. Most importantly, communities themselves could require the use of a PR approach for all research in which they participate.

### 4.4. Strengths and Limitations

This review has several strengths and some limitations. The article selection process was designed to promote replicability of results, consistency across reviewers, and a final dataset that was manageable in scope while also representative of the broader literature on health-related PR involving AIAN communities. Article selection and organization was guided by formal protocols that included detailed instructions and defined keywords and were field-tested to improve clarity and usability. Guidance documents describing best practices for article selection and the decision to have each article reviewed by two authors further contributed to the rigor of the article selection process.

The design of the data extraction form and the conduct of the extraction process was another strength of this study. All queries on the data extraction form included standardized response sets and inclusion/exclusion criteria and most queries also included the option to provide open-ended responses. Most importantly, queries were developed to reflect the principles and mechanisms of community-based participatory research and tribal participatory research—the dominant approaches to PR involving AIAN communities. Decisions about the relevance of specific data points to the research question are therefore grounded in theories of PR that have been developed over many years and that represent that best available scholarship on PR. A field-test of the data extraction form and weekly conference calls throughout the extraction process also contributed to consistency and replicability.

Despite these strengths, opportunities for improving the data extraction form and extraction processes exist. First, aligning queries on the data extraction form with the principles of dominant approaches to PR means that any shortcoming in these approaches will be represented in the data extraction form and the final dataset. This concern is especially relevant for research involving AIAN communities, since many approaches to PR are informed by the values and practices of Western science and culture and may discount aspects of the research process that are of import to AIAN peoples. While the data extraction form included queries designed to collect information of particular relevance to AIAN communities participating in PR, it is unlikely that all such information was extracted. Second, the large number of articles to be extracted meant that each article could be extracted only once. Although field-testing provided authors with an opportunity to directly compare a limited number of results and weekly conference calls helped ensure they were interpreting and responding to queries in the same way, the lack of a second extraction for each article precluded comparison of reviewer responses for the full dataset.

The adaptability of PR approaches constitutes a major methodological strength, but it also creates challenges for assessing adherence to PR principles. The nature and extent of community engagement in research processes will differ across PR projects according to the abilities and interests of the communities involved. Not every community will be interested in engaging, or even be able to engage, in all processes activities and communities will differ in the process and activities that they prioritize for engagement. As a result, judgments about the extent to which a given project adheres to the principles of the PR framework it employed cannot be made solely on the basis of information about the kinds of research activities in which community members were engaged. Lacking additional information about the processes and activities in which a community actually desired and intended to engage, it is not possible to directly assess the quality of PR projects in terms of their adherence to PR frameworks. For these reasons, the results of this study are strictly descriptive and do not include judgments about the quality of PR projects involving AIAN communities.

Another limitation of this dataset is that it reflects research activities as they were reported in articles, which may differ in important ways from the research activities that actually took place. Not every article about a PR project has reporting of community engagement in research processes as a primary objective and may not include details about these activities for that reason. This is common in cases where a single research project is described in multiple articles, only one of which provides a description and/or evaluation of research processes. Even among articles that do intend to describe community engagement activities, word limits may preclude the inclusion of all relevant details. This issue is complicated by the lack of standards for reporting on community engagement in PR, which creates problems for assessing the quality of PR and the extent to which a given project adheres to the principles of the PR approach it employed. The inability to distinguish between lapses in the application of the principles of PR and gaps in reporting on PR is a major limitation of this study and may have caused the results to underestimate or misrepresent the community engagement that actually occurred.

## 5. Conclusions

The purpose of this review was to characterize the state of health-related PR involving AIAN communities. Starting from an initial library of 4255 articles, we used a formal filtering process to identify 178 relevant articles published since January 1st, 2000. Using a data extraction form with 60 queries related to the principles and mechanisms of dominant approaches to PR, we collected more than 10,000 standardized and open-ended responses. The resulting dataset, rich in contextual detail and substantial in scope, admits of a wide range of analyses and represents a significant contribution to research in this area.

For this initial analysis, we provided a brief overview of the dataset before focusing on four areas of particular relevance to PR involving AIAN communities: community engagement in the research process; community guidance and tribal regulation of research; cultural adaptation; and, community research capacity. We have recommended several lines of inquiry for future research in this area and proposed changes in the practices and policies informing PR. Studies on the comparative effectiveness of different approaches to PR; improvements in study design; documents to guide conduct and reporting on PR; and, changes to grant timelines and budgets that account for the unique demands of PR have been proposed as strategies for maximizing the benefits future PR projects in AIAN communities.

## Figures and Tables

**Table 1:** Health Conditions/Issues of Interest in Articles Utilizing PR in AIAN Communities.

Health Conditions/Issues or Topics of Interest	Articles *n* (%)
Any Cancer	33 (18)
Breast Cancer ^1^	13 (7)
Cervical Cancer ^2^	6 (3)
Colorectal Cancer	2 (1)
Cancer (General/Other) ^3^	15 (8)
Any Mental/Behavioral Health	24 (13)
Historical Trauma	3 (2)
Suicide	13 (7)
Mental/Behavioral Health (General)	9 (5)
Any Substance Use/Misuse	34 (19)
Alcohol Misuse	13 (7)
Tobacco Use ^4^	8 (4)
Substance Misuse (General/Other)	16 (9)
CVD	4 (2)
Diabetes/Obesity	18 (10)
Elder Care	6 (3)
Environmental Health	8 (4)
Exercise/Diet	14 (8)
Family Wellness	9 (5)
Sexual Health ^5^	14 (8)
Evaluating/Developing Health Research Systems and Approaches	23 (13)
Evaluating/Developing Health Care and Public Health Systems	18 (10)
Other	21 (12)

^1^ Includes mammography. ^2^ Includes cervical health. ^3^ Includes cancer care. ^4^ Includes e-cigarettes. ^5^ Includes AIDS/HIV and sexually transmitted infections.

**Table 2:** Community Engagement in the Research Process.

Study Component	Example Activity	Y (%)	N (%)	NR (%)	NA (%)
Initiation	Ft. Peck Tribes invited Montana State University Staff to Ft. Peck Reservation to discuss potential research project on the community’s sexual and reproductive health needs [23].	49 (27)	20 (11)	110 (61)	1 (1)
Funding	Community members helped review and provide feedback on grant proposals and assisted in drafting grant proposals [24].	46 (26)	11 (6)	120 (67)	3 (2)
Research Question, Study Aims	Community elders, oral historian, and Lakota linguist involved in discussion that determined study goals [25].	118 (66)	6 (3)	55 (31)	1 (1)
Administration	Community researchers participated in selection, interview, and hiring of community organizers [26].	96 (53)	2 (1)	81 (45)	1 (1)
Design, Methods, Approach	Community input guided selection of screening methods and processes and targets related to adaptation of screening methods [27].	114 (63)	3 (2)	62 (34)	1 (1)
Protocols	CAG ^1^, community-based project staff, and researchers co-developed telephone scripts and surveys with community member input [28].	129 (72)	1 (1)	47 (26)	3 (2)
Meetings and Events	CAG meetings, open community meetings, executive board meetings, and monthly meetings between lay health advisors and project staff [29].	136 (76)	0 (0)	16 (9)	28 (16)
Data Collection	Community health representatives moderated focus groups and took notes [30].	115 (64)	7 (4)	56 (31)	2 (1)
Data Analysis	Community members helped develop codebook and were involved in coding and review of themes developed from codes [31].	83 (46)	14 (8)	77 (43)	6 (3)
Intervention	CAG suggested cultural adaptations to educational materials to promote organ donation, including addition of storytelling components, emphasis on community values of generosity, and local photography [32].	111 (62)	1 (1)	29 (16)	39 (22)
Dissemination	CAG members reviewed manuscripts draft and their comments were incorporated into final draft; CAG members also represented the project at national forums [33].	93 (52)	1 (1)	77 (43)	9 (5)

^1^ CAG = community advisory group.

**Table 3:** Community-Level Mechanisms for Guiding and Regulating Research.

Regulatory/Guidance Mechanism	Articles n (%)
Regulatory/Guidance Group CAG ^1^	125 (69)
Tribal Leader	11 (6)
Tribal Liaison/Facilitator	64 (36)
Community Leader	4 (2)
Tribal Council	45 (25)
Tribal Research Committee/Board	14 (8)
Tribal Health Committee/Board	18 (10)
Other Tribal Committee/Board	26 (14)
Tribal IRB ^2^	18 (10)
Regulatory/Guidance Document	
Research Code/Agreement	5 (3)
Memorandum of Agreement	10 (6)
Tribal Resolution	17 (9)
Data Sharing Agreement	4 (2)
Total Regulatory/Guidance Groups and Documents per Study	
0 Groups and Documents	21 (12)
1 Groups and Documents	44 (24)
2 Groups and Documents	56 (31)
3 Groups and Documents	38 (21)
4 Groups and Documents	12 (7)
>4 Groups and Documents	7 (4)

^1^ CAG = Community Advisory Group; ^2^ IRB = Institutional Review Board.

**Table 4:** Type and Number of Culturally Adapted Study Components.

Cultural Adaptation	Articles *n* (%)
Type of Cultural Adaptation	
Culturally Adapted Study Design	72 (40)
Culturally Adapted Study Protocols	109 (61)
Culturally Adapted Interventions	80 (44)
Dissemination Products and Methods	35 (19)
Number of Culturally Adapted Study Components	
0 Culturally Adapted Components	32 (18)
1 Culturally Adapted Components	55 (31)
2 Culturally Adapted Components	50 (28)
3 Culturally Adapted Components	23 (13)
4 Culturally Adapted Components	18 (10)

**Table 5:** Community Research Capacity and Co-learning.

Measures of Research Capacity or Co-learning	Y (%)	N (%)	NR (%)	NA (%)
Community members received research training	93 (52)	10 (6)	76 (42)	1 (1)
Community members received academic credit for research training	4 (2)	2 (1)	87 (48)	0 (0)
Community members were employed by researchers	50 (28)	7 (4)	120 (67)	3 (2)
Community had prior experience with research	82 (46)	1 (1)	96 (53)	1 (1)
Community members received training in PR	43 (24)	1 (1)	135 (75)	1 (1)
Researchers Received Training in Community Culture/History	60 (33)	0 (0)	117 (65)	3 (2)
Researchers had Prior Experience with Community	89 (49)	4 (2)	85 (47)	2 (1)
Researchers had or Received Training in PR	47 (26)	1 (1)	131 (73)	1 (1)

PR = Participatory Research.
